# A Straightforward Method to Produce Multi-Nanodrug Delivery Systems for Transdermal/Tympanic Patches Using Electrospinning and Electrospray

**DOI:** 10.3390/polym15173494

**Published:** 2023-08-22

**Authors:** Bahareh Azimi, Claudio Ricci, Teresa Macchi, Cemre Günday, Sara Munafò, Homa Maleki, Federico Pratesi, Veronika Tempesti, Caterina Cristallini, Luca Bruschini, Andrea Lazzeri, Serena Danti, Nazende Günday-Türeli

**Affiliations:** 1 Department of Civil and Industrial Engineering, University of Pisa, Largo L. Lazzarino 2, 56122 Pisa, Italy; 2 Consorzio Interuniversitario Nazionale per la Scienza e Tecnologia dei Materiali (INSTM), via G. Giusti 9, 50121 Florence, Italy; 3 Department of Translational Researches and New Technologies in Medicine and Surgery, via Savi 10, 56126 Pisa, Italy; 4 MyBiotech GmbH, Industriestrasse 1B, 66802 Überherrn, Germany; 5 Department of Carpet, Faculty of Arts, University of Birjand, Birjand 9717434765, Iran; 6 Institute for Chemical and Physical Processes (IPCF), National Council of Researches (CNR), via G. Moruzzi 1, 56124 Pisa, Italy; 7 Department of Surgical, Medical, Molecular Pathology and Emergency Medicine, University of Pisa, via Savi 10, 56126 Pisa, Italy

**Keywords:** fibers, nanoparticles, poly(hydroxybutyrate-*co*-hydroxyvalerate) (PHBHV), poly(lactic-glycolic acid) (PLGA), controlled drug release, dexamethasone, rhodamine, human dermal fibroblasts, eardrum, bio-based

## Abstract

The delivery of drugs through the skin barrier at a predetermined rate is the aim of transdermal drug delivery systems (TDDSs). However, so far, TDDS has not fully attained its potential as an alternative to hypodermic injections and oral delivery. In this study, we presented a proof of concept of a dual drug-loaded patch made of nanoparticles (NPs) and ultrafine fibers fabricated by using one equipment, i.e., the electrospinning apparatus. Such NP/fiber systems can be useful to release drugs locally through the skin and the tympanic membrane. Briefly, dexamethasone (DEX)-loaded poly(3-hydroxybutyrate-*co*-3-hydroxyvalerate) (PHBHV) fiber meshes were decorated with rhodamine (RHO)-loaded poly(lactic-*co*-glycolic acid) (PLGA) NPs, with RHO representing as a second drug model. By properly tuning the working parameters of electrospinning, DEX-loaded PHBHV fibers (i.e., by electrospinning mode) and RHO-loaded PLGA NPs (i.e., by electrospray mode) were successfully prepared and straightforwardly assembled to form a TDDS patch, which was characterized via Fourier transform infrared spectroscopy and dynamometry. The patch was then tested in vitro using human dermal fibroblasts (HDFs). The incorporation of DEX significantly reduced the fiber mesh stiffness. In vitro tests showed that HDFs were viable for 8 days in contact with drug-loaded samples, and significant signs of cytotoxicity were not highlighted. Finally, thanks to a beaded structure of the fibers, a controlled release of DEX from the electrospun patch was obtained over 4 weeks, which may accomplish the therapeutic objective of a local, sustained and prolonged anti-inflammatory action of a TDDS, as is requested in chronic inflammatory conditions, and other pathological conditions, such as in sudden sensorineural hearing loss treatment.

## 1. Introduction

The search for topical delivery systems able to release drugs by crossing the body barriers is recently becoming a hot field of pharmaceutical study. The outer surface of our body is largely delimited by the skin. At a smaller but significant extent, the eardrum likewise delimits the outer ear. Skin and eardrum share relevant similitudes: both are mechanically sensing systems, act as natural defense barriers against foreign damaging agents and the microbial invasion of the tissues underneath, are in fact covered by squamous epithelia, and are affected by wounds that sometimes cannot self-heal, such as ulcers and tympanic perforations [1]. In addition, both skin and eardrum offer a way for the topical delivery of bioactive agents needed by skin and middle ear pathology, usually anti-inflammatory molecules, antibiotics and growth factors, beyond the barrier, using biocompatible polymers. Polymeric materials can indeed be processed to have films or textures suitable as patches.

Among the many polymers, polyhydroxyalkonates (PHAs) are a novel class of biodegradable and biocompatible polyesters produced by various microorganisms, thus representing a promising green polymer source obtained via biotechnology routes. These polymers have attracted much attention in several fields, including the biomedical market, which has recently started to replace petroleum-derived products to improve biocompatibility [2,3]. In fact, the broad range of mechanical and biological properties of PHAs make them excellent and versatile candidates for biomedical applications [4]. A variety of PHAs with diverse characteristics can be created by applying different fermentation process conditions [5]. Among others, poly(3-hydroxybutyrate-*co*-3-hydroxyvalerate) (PHBHV), obtained via the copolymerization of poly(3-hydroxybutyrate) P(3HB) with 3-hydroxyvalerate (3HV), is a well-known member of the PHA family. PHBHV shows a lower crystallinity, greater polymer chain flexibility, and easier processability than P(3HB), and is thus appealing for soft tissue interactions [6,7]. Its flexible polymer chains make PHBHV suitable to be processed into fiber structures. In this view, the electrospinning process is a unique method to produce fibrous meshes with fiber diameters ranging from a few microns to tens of nanometers [8]. It is generally considered a promising technique for the fabrication of tissue-engineered scaffolds, since the fibrillar part of the tissue extracellular matrix (ECM), which plays a key role in cell adhesion, migration and function, is made up of fibers with a similar morphology and size [2,9]. Moreover, the extremely high surface area-to-volume ratio of the electrospun fibers and the resulting highly porous structure of the mesh offer exploitable features for several applications [10].

Electrospun fibrous meshes can also be used in conventional transdermal drug delivery systems (TDDs) and transdermal patches, since they can carry a wide range of pharmaceutical ingredients, including antitumor drugs, antibiotics, anti-inflammatory drugs, nucleic acids and proteins [11]. The mechanisms of drug release can be adjusted by modifying the electrospinning parameters and, consequently, fiber properties [12,13]. In addition, the porous structure of the electrospun fibrous mesh can allow an appropriate release of bioactive molecules from the polymeric matrix [14]. Finally, the systemic absorption of the drug is reduced by the topical distribution obtained in electrospun fibers, which results in a lower dosage than using pills and injections [15]. Topical drug delivery via drug-eluting electrospun patches brings the beneficial effects of decreasing dose frequency and avoiding primary drug metabolism in the liver by delivering drugs directly to the desired body sites. Drug-eluting patches are currently applied for the administration of analgesic, hormonal or anti-inflammatory drugs through the skin [16]. However, the application of electrospun fibers as TDD skin patches has received less attention in comparison to implantable scaffolds or wound dressings. Anyway, electrospun fiber patches are entitled with some advantages, which are significant in this specific case. Firstly, the upscaling of PHA products, particularly in the biomedical sector, can be promoted by the electrospinning technique due to the reduced polymer quantity needed to obtain such porous and nanofibrous nonwovens. Since biotechnology processes are more expensive than conventional polymer production processes, PHAs hold high potential to develop sustainable products in the biomedical field, which operates on lower volumes and higher quality [17]. Secondly, the process is usually performed at room temperature, which saves energy consumption with respect to molten processes used for producing films, and the solvents can be theoretically recovered and recycled. Lastly, fibrous patches offer a higher surface for drug delivery and cell growth, irrespective of flat film patches [18]. To enable a multi-drug delivery, drug-loaded nanoparticles (NPs) can be applied. Conventional technologies to produce NPs are based on nanoemulsions, whereas emerging non-conventional technologies are electrospray and micro/nanofluidic systems. Electrospray is also performed using an electrospinning apparatus under a different operating mode. A low polymer concentration in the solution, along with other variations in parameters, allows particles and fibrils often in micro- or nano-metric ranges to be generated [19]. In the electrospray system, the charged liquid jet, at some point, breaks up into droplets. During their flight to the collector, the solvent evaporation makes the primary droplets shrink, which leads to the increase in charge concentration, so the primary droplets finally will break up into smaller offspring [20]. Electrospray technique has been used to produce different synthetic and natural polymeric NPs, such as poly(lactic-*co*-glycolic acid) (PLGA) and polysaccharides [21,22,23]. The surface functionalization materials are also possible by using the electrospray method [24].

**Figure 1:**
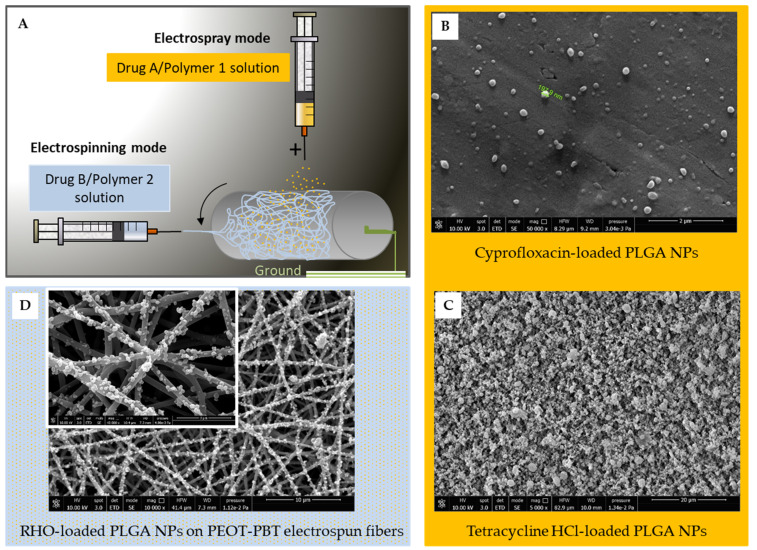
Examples of the versatility of electrospray/electrospinning system with different drug/PLGA NPs produced via electrospray, also in combination with electrospun fiber meshes: (**A**) schematic of the used process; (**B**) Ciprofloxacin-loaded PLGA NPs (~200 nm), electrosprayed for a short time (50,000× magnification; 10 kV; scale bar 2 µm); (**C**) Tetracycline hydrochloride (HCl)-loaded PLGA NPs (5000× magnification; 10 kV; scale bar 20 µm); (**D**) RHO-loaded PLGA NPs deposited onto PEOT-PBT electrospun fibers (10,000× magnification; 10 kV; scale bar is 10 µm); lens (40,000× magnification; 10 kV; scale bar is 3 µm). (Images are from the authors).

The aim of this study was to set up a method based on the electrospinning apparatus, for an all-in-one production of a multi-nanodrug eluting nonwoven, as a potential TDDS useful as a skin or tympanic patch. As a proof of concept, we designed and characterized a dual-nanodrug system. Ultrafine PHBHV fibers were loaded with dexamethasone (DEX) as an anti-inflammatory drug, and the DEX-loaded PHBHV fibers were decorated with PLGA NPs containing rhodamine (RHO) as a second drug model, obtained via electrospray. In this way, we propose a method for a multi-nanodrug-eluting patch for local drug delivery applications, which can be potentially extended to different (co-)polymers and drugs (Figure 1).

As an example, other fiber meshes, e.g., produced using poly(ethylene oxide terephthalate-*co*-butylene terephthalate) (PEOT-PBT), can be combined with RHO-loaded PLGA NPs, and PLGA NPs can be prepared by incorporating not only RHO, or also antibiotics, such as Ciprofloxacin and Tetracycline–HCl (Figure 1).

In this study, we specifically investigated a TDDS based on DEX-loaded PHBHV fibers and RHO-loaded PLGA NPs. We assessed the presence of NPs adhered to the PHBHV fibers, the mechanical properties of the fiber mesh and the drug release profiles of the nonwovens. The in vitro release studies included DEX from PHBHV fibers and RHO from PLGA NPs, alone and within the fully assembled nonwoven, up to 4 weeks. Cytocompatibility of the drug-loaded samples was investigated using human dermal fibroblasts (HDFs).

Due to its slow biodegradation, the use of PHBHV for the fibers allows for a long-lasting release profile of DEX, a powerful anti-inflammatory agent, thus acting on both acute and chronic inflammation responses. New biomedical routes are recently approaching bio-based materials and low environmental impact technologies, in terms of reducing solvents, energy consumption and chemical waste, increasing process yield and improving therapy efficacy. The NP-functionalized DEX-loaded PHBHV fiber patches are in line with this vision, as they would enable the development of novel bio-based products for tissue engineering and drug delivery, with a straightforward high-yield method using a reduced amount of solvents.

## 2. Materials and Methods

### 2.1. Materials

Poly(3-hydroxybutyrate-*co*-3-hydroxyvalerate) (PHBHV; code 403121), poly(d,l-lactide-*co*-glycolide) (PLGA; code 719870), chloroform (code 102442), methanol (code 34860), dichloromethane (code 32222), acetonitrile (ACN; code 34851), dexamethasone (DEX; code D4902), rhodamine (RHO; code 83689), l-glutamine (code G7513), penicillin/streptomycin (Pen-Strep; code P0781), trypsin-EDTA solution (code T2610) and resazurin sodium salt (code R7017) were purchased from Sigma-Aldrich (Milan, Italy). Diflucan (infusion solution, 2 mg/mL) was purchased from Pfizer Inc. (New York, NY, USA), while Levofloxacin (infusion solution, 5 mg/mL) was purchased from Fresenius Kabi Italia s.r.l. (Isola della Scala, VR, Italy). Dulbecco’s modified Eagle’s medium (DMEM; code 11880-028), fetal bovine serum (FBS; code 10500064), phosphate-buffered saline 1×, (PBS; code 14190169), and Calcein AM were purchased from Gibco (by Life Technologies ThermoFisher Scientific, Waltham, MA, USA). Collagenase I was obtained from Worthington Biochemical Corp. (Lakewood, NJ, USA).

### 2.2. Production of RHO-Loaded PLGA NPs

PLGA was dissolved in dichloromethane/methanol (4:1 *w*/*w*) solution at a polymer/solvent concentration of 0.5% *w*/*v*% and stirred at 300 revolutions per minute (rpm) for 12 h at room temperature (RT). To produce RHO-loaded PLGA particles, 1.15% (RHO/PLGA *w*/*w*%) was added to the solution and stirred at 300 rpm for 12 h at RT. The polymer solution was loaded into a 10 mL glass syringe with a blunt tip stainless steel needle (21G × 3/4”) and placed in a syringe pump (NE-300, New Era Pump Systems, Inc., Farmingdale, NY, USA) within an electrospinning apparatus (Starter kit; Linari Engineering s.r.l., Pisa, Italy). A static collector (grounded) covered with an aluminum foil was placed at a 28 cm distance from the tip of the positive polarity needle and 40 kV potential were applied (S1600079 Linari High Voltage, Linari Engineering s.r.l.). The polymer solution (5 mL, leading to 25 mg of PLGA) was injected at a constant flow rate of 60 µL/h. All the fabrication steps were performed at RT with relative humidity (RH) of 35%.

### 2.3. Production of DEX-PHBHV Electrospun Fiber Meshes with RHO-PLGA NPs

PHBHV was dissolved in chloroform/methanol (9:1 *w*/*w*) mixture at a polymer/solvent concentration of 15% *w*/*w*% and stirred at 300 rpm for 12 h at RT. To obtain DEX-loaded PHBHV fibers, DEX at 10% (DEX/PHBHV *w*/*w*%) was added to the solution and stirred at 300 rpm for 12 h RT. The polymer solution was loaded into a 10 mL glass syringe, fitted with a blunt tip stainless steel needle (21G × 3/4”), and placed into a syringe pump (NE-300). The ground terminal of high voltage supply was connected to the metal needle, while the positive terminal was connected to a cylindrical collector (Easydrum; diameter 8 cm, Linari Engineering s.r.l.) placed at 15 cm distance of from the tip of the needle. Therefore, 30 kV potential was applied. The polymeric solution was injected from the needle in the presence of the electric field at a constant flow rate of 0.001 mL/min. All the fabrication steps were performed at RT with an RH of 46%.

To produce the dual drug-loaded nonwoven, RHO-loaded PLGA NPs were electrosprayed onto the previously produced DEX-loaded PHBHV fibers mesh using the same electrospinning apparatus and the working and environmental conditions reported above (Section 2.2) for 60 min. After production, all the samples were vented overnight to remove any putative traces of solvents.

### 2.4. Morphological, Chemical and Mechanical Characterization

The morphology of the NPs and the fiber meshes was assessed under scanning electron microscopy (SEM) (FEI FEG-Quanta 450 instrument, Field Electron and Ion Company, Hillsboro, OR, USA). The samples were sputtered with gold (Gold Edwards SP150B, UK) before analysis. ImageJ software (version 1.46 r; http://imagej.nih.gov, accessed on 2 May 2023) was used to determine the average diameter of NPs and, fibers, as well as average mesh size obtained from SEM micrographs (for each analysis, *n* = 100). Mesh size was calculated as pore equivalent diameter [24].

Fourier transform infrared spectroscopy (FTIR; Nicolet T380, Thermo Scientific, Waltham, MA, USA) under attenuated total reflectance (ATR) mode was used to analyze the chemical structure of the samples. An average value of 128 scans at 4 cm^−1^ resolutions was collected for each sample.

The mechanical behavior of the fiber meshes (*n* = 3) was investigated via a dynamometer INSTRON 5500R equipped with MERLIN software V.4.42 (INSTRON, Norwood, MA, USA). We used a load cell of 10 N maximum capacity and a crosshead speed of 5 mm/min. To enhance the stability of the fiber mesh in the positioning phase, a paper frame was employed. The gauge length of the electrospun fiber meshes was set at 2 cm, determined by measuring the distance between the parallel strips of the frame. After positioning, the paper frame was cut at the sides. The cross-sectional area of the fiber mesh was obtained through measurement with a thickness gauge.

### 2.5. Drug Loading and Entrapment Efficiency

The drug loading percent (*DL*%) and encapsulation efficiency (*EE*%) were calculated using the following formulas [25]:

(1) $$DL\%=\frac{mass\;drug\;loaded\;in\;NPs}{mass\;NPs}\times 100$$

To calculate (*DL*%, lyophilized NPs were weighed and dissolved in acetonitrile (ACN). Drug content was measured by HPLC-UV (Hitachi Chromaster^®^ equipped with Chromaster 5410 UV Detector, VWR, Darmstadt, Germany) method described in Section 2.6.

(2) $$EE\%=\frac{actual\;drug\;loading}{theoretical\;drug\;loading}\times 100$$

To calculate *EE*%, NPs were dissolved using ACN and centrifuged at 13,000× *g* for 45 min and the supernatants were diluted in ACN and measured by HPLC-UV (Hitachi Chromaster^®^ equipped with Chromaster 5410 UV Detector, VWR, Radnor, PA, USA) method described in Section 2.6. For percentage calculations, a quantity of NPs or fibers was weighted (e.g., ~5.2 µg of NPs or ~6.8 mg of fiber mesh) and the theoretical DEX and RHO content was calculated based on the drug/copolymer ratio (*w*/*w*%) used during the fabrication processes. Plain PHBHV fibers were used as negative controls for DEX or RHO release quantification. Due to the high efficiency of the electrospinning/electrospray process, the theoretical drug loading is considered to be the same of the drug added to the copolymer solution.

### 2.6. DEX and RHO Release from PHBHV Meshes and NPs

Release studies were performed for RHO-loaded PLGA NPs and PHBHV fibers containing RHO-loaded PLGA NPs, as well as DEX-loaded PHBHV fibers and DEX-loaded PHBHV fibers containing RHO-loaded PLGA NPs up to 24 h. Moreover, DEX release was further monitored for 28 days. For release kinetics studies, fiber meshes (6–8 mg) were suspended in 2.5 mL PBS pH 7.4 at 37 °C (*n* = 3). At predetermined time intervals, 100 µL samples were collected for the quantification of released DEX and/or RHO, which was immediately replaced with fresh buffer. Spectrophotometric assay analysis of RHO was performed by using UV–Vis photometer set at 533 nm. The spectrophotometric method was shown to be linear (y = mx) for a range of 0.2–100 µg/mL. A validated high-performance liquid chromatography (HPLC) (Hitachi, Chromaster, Germany) was used to determine the release of DEX in PHBHV fibers. Release tests were performed in PBS pH 7.4 at 37 °C under constant stirring. Samples were measured without any treatment prior to analysis. HPLC analysis was conducted using column Gemini C18 Phenomenex (4.6 mm × 100 mm, 3 µm 110 Å). The mobile phase was ACN/water (30/70) at a flow rate of 1.3 mL/min. Injection volume set to 20 µL DEX was detected at 254 nm. The chromatograms were analyzed via OpenLab software. HPLC was also used to determine the release of DEX form electrospun meshes up to 28 days. The released kinetics was reported as the cumulative percent of drug released in comparison to loaded drug vs. time.

### 2.7. Ethical Statement

HDFs were isolated from waste samples of normal skin derived from contralateral mastectomy of adult women, otherwise destined to be disposed of. The collected samples were mixed biowaste. They were anonymous samples and were treated in conformity with the Declaration of Helsinki.

### 2.8. HDF Isolation and Culture

Skin samples were washed with sterile PBS supplemented with penicillin/streptomycin and cut into 2–3 mm pieces. Skin pieces were then incubated for 18 h with DMEM, 5 mM CaCl_2_ and 0.25% (*v*/*v*%) collagenase I enzyme. After digestion, EDTA was added to neutralize enzymatic activity. Cell suspension was then washed two times with 10% (*v*/*v*%) FBS DMEM and cultured in a complete culture medium, consisting of DMEM with an added 10% (*v*/*v*%) of FBS, 2 mM L-glutamine, 100 IU/mL penicillin and 100 mg/mL streptomycin. On passage 3, HDFs were characterized by FACS analysis, evaluating the expression of fibroblastic markers, such as CD90-, Thy1 and cadherin-11. Cell culture was performed in complete culture medium until 80–90% confluence, in humidified 5% CO_2_/95% air incubator set at 37 °C. HDFs were used at passage 6 for the cytocompatibility experiments.

### 2.9. Cytocompatibility

The nonwoven samples (*n* = 3) were puncher-cut in 10 mm discs, sterilized by UV irradiation for 30 min for each side and placed in 24-well plates prior to cell seeding. HDFs were expanded in HDF culture medium. Therefore, HDFs were trypsinized and seeded onto each nonwoven sample at a density of 250,000 cells in 33 μL of culture medium. The samples were placed in a cell culture incubator. After this time, 1 mL of culture medium, with an added 1% (*v*/*v*%) Diflucan and 1% (*v*/*v*%) Levofloxacin, was poured into each well to prevent contamination. The culture was carried out for 8 days, replacing the culture medium every 2–3 days.

Resazurin dye assay was used to assess the metabolic activity during the culture. Resazurin turns from blue into pink because of cellular reduction. Briefly, resazurin sodium salt was dissolved at 0.5 mg/mL in PBS and sterile filtered to obtain a stock solution. At each time-point, the culture medium was removed, replaced with culture medium containing 20 μL/mL resazurin solution and left to incubate. After 3 h, the supernatant was removed and read in a plate reader (Victor 3; PerkinElmer, Waltham, MA, USA) using a double wavelength reading, at 570 nm and 600 nm, respectively. The percentage of dye reduction against non-cellularized controls was calculated by considering the molar extinction coefficients at the two wavelengths. The samples were assayed in triplicate (*n* = 3) and each sample was run 3 times. Data were given as mean ± standard deviation.

At the endpoint, the culture medium was removed, and the samples were incubated with Calcein AM at 1 μL/mL in sterile PBS in the dark for 20 min. Viable cell cytoplasm is stained in green. Fluorescence analysis was carried out using an inverted microscope equipped with a FITC fluorescein filter (Nikon Eclipse Ti, Nikon, Tokyo, Japan).

### 2.10. Statistical Analysis

Statistical analysis was performed using SPSS software (v.16.0; IBM, Armonk, NY, USA). Data were processed using one-way analysis of variance (ANOVA) and post hoc test (Duncan) for multiple comparisons. Probability (*p*) values < 0.05 were set as statistically significant.

## 3. Results

### 3.1. Morphological and Dimensional Characterization of NPs

**Figure 2:**
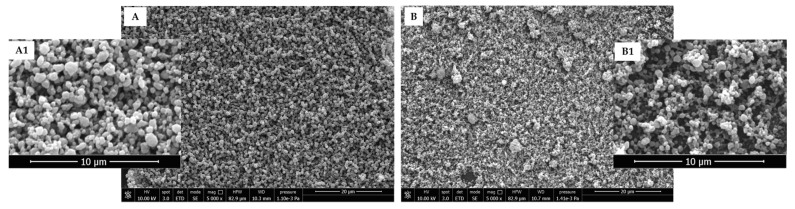
Representative SEM micrographs of PLGA NPs obtained via electrospray (**A**) plain PLGA NPs; (**B**) RHO-loaded PLGA NPs. SEM analysis was performed by applying 10 kV and 5000× original magnification, scale bar is 20 µm. (**A1**,**B1**) lens showing zoomed-in images related to PLGA and RHO-PLGA NPs, respectively.

The morphology of plain PLGA NPs is visible in Figure 2A,A1. The SEM micrographs showed that smooth and almost spherical particles were produced with an average size of 774 ± 182 nm. SEM analysis clearly indicated that no hairline cracks or heterogeneity were found on the surface of these NPs. The morphology of RHO-loaded PLGA NPs is shown in Figure 2B,B1.

The presence of RHO decreased the size of NPs down to 303 ± 89 nm and led to the creation of some small clusters with an average diameter of 2.61 ± 0.62 µm.

### 3.2. Morphological and Dimensional Characterization of the Fiber Meshes

**Figure 3:**
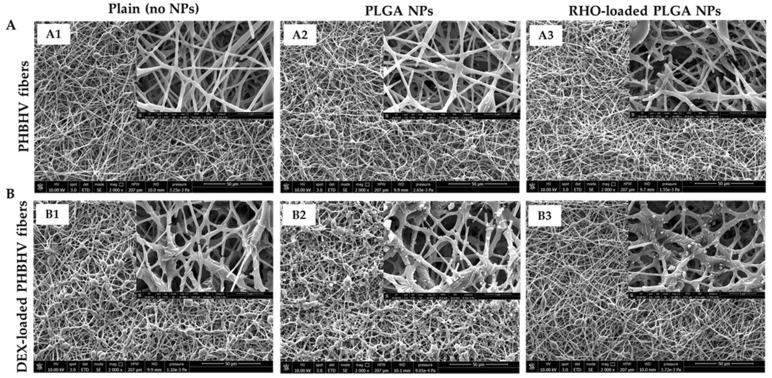
SEM micrographs showing electrospun PHBHV fibers: (**A**) plain (unloaded), and (**B**) DEX-loaded. (**A1**,**B1**) Plain and DEX-loaded PHBHV fibers. (**A2**,**B2**) Plain and DEX-loaded PHBHV fibers with PLGA NPs. (**A3**,**B3**) Plain and DEX-loaded PHBHV fibers with RHO-loaded PLGA NPs. (**A**,**B**) 2000× magnification; 10 kV; scale bar is 50 µm; Lenses: 16,000× magnification; 10 kV; scale bar is 5 µm.

The morphology and size of electrospun fibers were evaluated by SEM and ImageJ (Version 1.53t) software (Figure 3).

Beaded PHBHV fibers were observed in all samples. PHBHV fibers with a smooth surface and fiber size of 935 ± 230 nm were obtained by electrospinning the plain copolymer (Figure 3A). The average pore size of the mesh was 1.94 ± 0.78 µm. The addition of DEX (10% *w*/*w*%) increased the number of beads within the mesh structure (Figure 3B) and increased the fiber diameter up to 1106 ± 440 nm. The average pore size of the mesh was 2.35 ± 1.09 µm. Via electrospray, plain PLGA NPs (Figure 3A2,B2) and RHO-loaded PLGA NPs (Figure 3A3,B3) were successfully located at the surface of both plain and DEX-loaded PHBHV fibers. A higher density of NPs was detected on the surface of DEX-loaded PHBHV fibers (Figure 3B2,B3), in comparison to the unloaded ones.

### 3.3. Chemical Characterization

**Figure 4:**
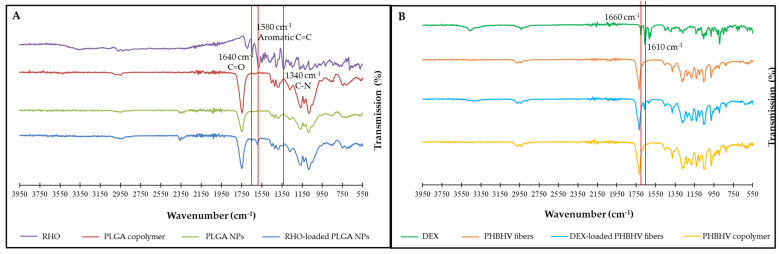
FTIR spectra of (**A**) RHO, PLGA copolymer, unloaded PLGA NPs and RHO-loaded PLGA NPs and (**B**) RHO-loaded PLGA NPs, indicating the presence of RHO within the NPs and the presence of DEX within the fiber meshes.

The presence of RHO within the electrosprayed PLGA NPs was checked by FTIR (Figure 4A). The spectra of RHO, PLGA copolymer, unloaded PLGA NPs and RHO-loaded PLGA NPs are shown in Figure 3A. Characteristic peaks of RHO at 1340 cm^−1^, 1580 cm^−1^ and 1640 cm^−1^ were observed in RHO-loaded PLGA NPs, indicating the presence of RHO within the NPs. FTIR analysis also confirmed the presence of DEX within the PHBHV electrospun fibers (Figure 4B). The spectra of DEX, PHBHV copolymer, unloaded PHBHV fibers and DEX-loaded PHBHV fibers meshes are shown in Figure 3B. The characteristic peak at 1660 cm^−1^ was observed in different DEX-loaded PHBHV electrospun samples, demonstrating the presence of DEX within the fibers.

### 3.4. Mechanical Characterization of the Fiber Meshes

**Figure 5:**
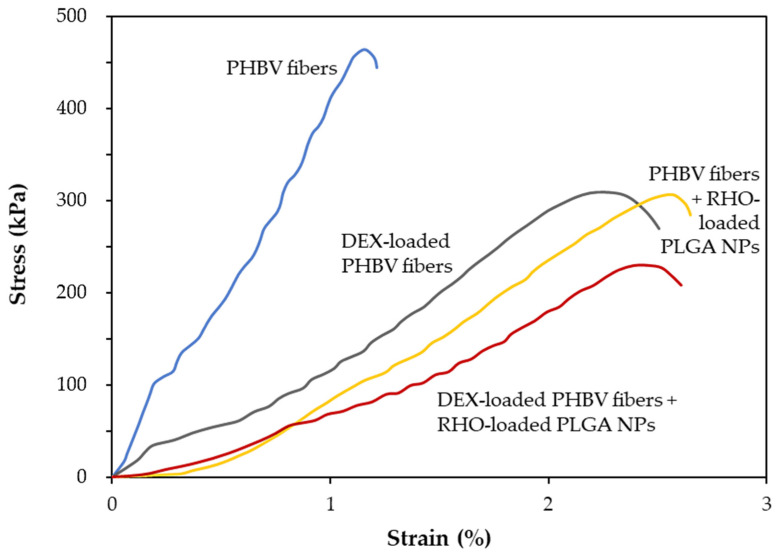
Representative stress–strain curves of PHBHV electrospun fiber meshes +/− DEX and +/− RHO NPs.

The effect of PLGA NPs and DEX on the mechanical properties of PHBHV electrospun fibers was investigated using a dynamometer INSTRON under tensile mode. The stress–strain curves obtained by testing different fibrous meshes are presented in Figure 5. Table 1 shows the Young’s modulus of different fiber meshes, which is considered the only reliable property obtained due to the slipping of some samples. The results highlighted that these meshes were mechanically soft; in fact, the Young’s moduli values were lower than 1 MPa (Table 1).

It is interesting to note that the fiber meshes incorporating DEX, as well as those electrosprayed with RHO-loaded NPs, showed an averagely reduced Young’s modulus with respect to the plain PHBHV fiber mesh, thus making the dual-drug loaded sample the least rigid and most elastic one among all the tested samples. By adding DEX, the Young’s modulus decreased by 3.5 times, from 0.65 MPa (plain PHBHV fiber mesh) to 0.18 MPa (DEX-loaded PHBHV fiber mesh) (*p* < 0.05). It further decreased down to 0.14 MPa (DEX-loaded PHBHV fiber mesh, added with RHO-loaded PLGA NPs) and to 0.10 MPa (plain PHBHV fiber mesh, added with RHO-loaded PLGA NPs) following electrospraying with RHO-loaded PLGA NPs. Statistical significance was only found comparing plain fibers with all the other types of fibers.

### 3.5. DEX and RHO In Vitro Releases

**Figure 6:**
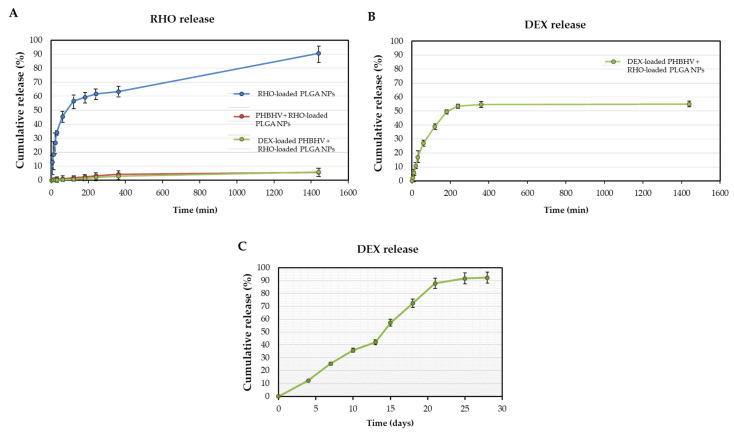
Cumulative release percentage of (**A**,**B**) in 24 h: (**A**) RHO from RHO-loaded NPs and RHO-loaded NP/fiber mesh (unloaded and DEX-loaded) and (**B**) DEX from RHO-loaded NP/fiber mesh; (**C**) in 28 days: DEX from DEX-loaded fiber meshes (without NPs). Measurements in graphs are reported as mean ± standard deviation (*n* = 3).

The in vitro releases of RHO from PLGA NPs and of DEX from the fiber mesh were evaluated over 1 day and 28 days, respectively (Figure 6).

RHO, incorporated inside the PLGA NPs via electrospray, resulted in a loading efficiency of 89%.

Figure 6A shows RHO, released from PLGA NPs and from PLGA NPs electrosprayed on PHBHV fibers, within the first 24 h. Approximately more than 50% of RHO was released from PLGA NPs in 4 h, and 90% in 24 h. The amount of RHO released was significantly decreased after 24 h (~5%) when PLGA NPs were located on the surface of PHBHV fibers (Figure 6A). It is observed that RHO released from the NPs was much lower than that obtained from the RHO-loaded NPs electrosprayed on the PHBHV fibers, independently of DEX loaded in the fibers (Figure 6A), because of the lower NP quantity deposited on the fibers.

The loading efficiency of DEX in PHBHV fiber mesh was 95%. An initial release (~11%) was observed within the first 4 days (Figure 6B). Then, a sustained release of DEX was observed over the following days, leading to 89% of DEX released in the investigated period (i.e., 28 days) (Figure 6C).

### 3.6. Cytocompatibility

**Figure 7:**
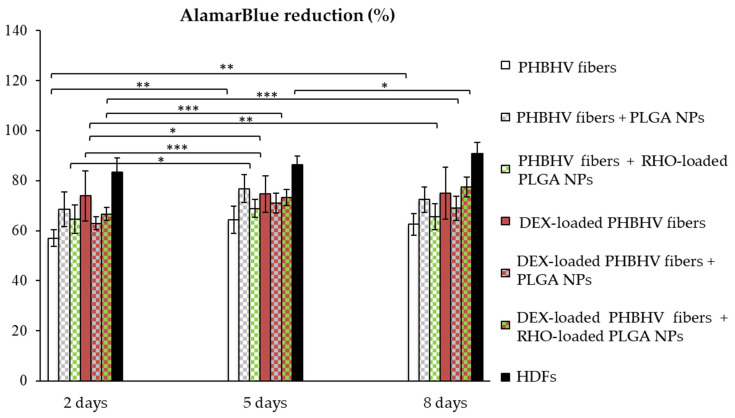
Metabolic activity of HDFs cultured on differently drug-loaded PHBHV fibers, performed via AlamarBlue assay (*n* = 3). Data are reported as mean ± standard deviation; asterisk shows statistical significance: * *p* < 0.05; ** *p* < 0.001; *** *p* < 0.0001.

**Figure 8:**
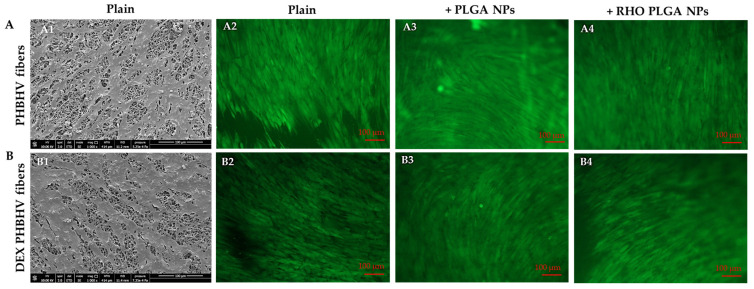
Imaging analyses on (**A**) plain and (**B**) DEX-loaded PHBHV fiber meshes, without and with electrosprayed NPs, namely (**A1**,**A2**,**B1**,**B2**) untreated, or (**A3**,**A4**,**B3**,**B4**) surface-decorated with (**A4**,**B4**) RHO-loaded and (**A3**,**B3**) unloaded PLGA NPs, after being cultured for 8 days with HDFs. (**A1**,**B1**) Results of SEM analysis; scale bar is 100 µm, voltage is 10 kV, 1000× magnification. (**A2**–**A4**,**B2**–**B4**) Results of Calcein AM fluorescent staining; scale bar is 100 µm.

The characterization of isolated HDFs is shown in Figure S1 (Supplementary). The cytocompatibility of all the produced fiber meshes namely, with/without the NPs and/or drug(s) was tested in vitro with HDFs, using a direct test (Figure 7 and Figure 8). HDF metabolic activity was evaluated at different time-points up to 8 days via AlamarBlue assay (Figure 7).

Statistical comparisons between HDFs cultured on plastics and those cultured on the different NP/fiber samples are reported in Table S1 (Supplementary). The presence of NPs, RHO and DEX showed an increased metabolic activity compared to plain PHBHV fiber meshes. For all the fibrous meshes, except for plain PHBHV at the earliest time-point, metabolic activity was always above 60%, resulting in either to a steady state or growing with time. A 10–15% decrease with respect to the positive control (only HDFs) was observed, due to the diverse adhesion substrate (fiber meshes or tissue culture plastics). The DEX-loaded PHBHV fiber mesh decorated with RHO-loaded PLGA NPs showed 77.4 ± 4.0% of dye reduction, being the highest among the fibrous meshes.

At the endpoint (i.e., on day 8), the samples were treated with Calcein AM and observed under an inverted microscope equipped with a fluorescence lamp (Figure 8A2–A4,B2–B4). After imaging, the samples were fixed in formalin, dehydrated and sputter-coated for observation via SEM (Figure 8A1,B1).

HDFs were able to adhere to the surface of the fiber meshes, both unloaded (Figure 8A) and DEX-loaded (Figure 8B), and to homogeneously cover the substrates. HDFs were viable and showed an elongated morphology. Qualitatively, no -relevant differences in cell morphology could be observed among the samples. Some small HDF-uncovered regions were detected mostly in plain PHBHV fiber meshes, whereas the fiber meshes decorated with PLGA NPs (both unloaded and RHO-loaded) were almost fully covered by HDFs after 8 days in culture.

## 4. Discussion

The presence of blood vessels and dendritic cells enables drugs to enter across the skin and eardrum barriers [26]. Conventional TDDS and transdermal patches have been designed for the delivery of drugs through the skin and can be used as a desired alternative to oral drug delivery. Besides the advantages of TDDs, such as dose fluctuation reduction, an improved efficacy of medications, stabilized drug diffusion profile and a greater bioavailability than systemic administration, only hydrophobic medications with low concentrations could successfully penetrate the depth of the skin, and the penetration of large hydrophilic drugs through the skin is still challenging [10]. Nanodrug delivery systems are recently increasing in popularity, as they show potential to be safer, more effective and require lower-cost drug formulations than micro- and macroscale counterparts [27].

Polymeric fibers produced by different spinning techniques have been used as carriers for different bioactive agents [28]. Electrospun fiber can be produced with remarkable properties, namely high surface area, surface nanopores, surface capability for physical and chemical activation as well as tailored physical and mechanical properties, which make them suitable nanosystems for drug or gene delivery; moreover, the fiber mesh shows macroscale cues by acting as a 3D system useful for wound dressing and tissue engineering [29]. The three-dimensional (3D) porous structure deriving from the arrangement into highly dense ultrafine fiber or nanofiber meshes allows molecules to be released from the polymer matrix with distinct release features [10]. Another explored way for nanodrug delivery is through NPs; therefore, the combination of nanofibers with NPs can be a versatile method to assemble diverse nanodrug delivery systems. There are different technologies that enable the combination of nano/ultrafine fibers and NPs, including the electrospray of suspensions of NPs prepared and pre-loaded by means of other technologies, such as nanoprecipitation or emulsions [23,27,30].

In our study, we investigated a single technology, which inherently permits the production of drug-loaded nano/ultrafine fibers as well as NPs, namely electrospinning. According to the density of the solutions and other working and environmental parameters, it is possible to produce fiber meshes, decorated with NPs, each made of a different polymer and loaded with different drugs. Ideally, if more pumps are applied on the same set-up, multi nanodrug systems can be developed. In our study, PHBHV was chosen to develop a transdermal/tympanic patch able to carry DEX and release it in a controlled manner. PHAs are highly biocompatible green polyesters, produced by bacterial fermentation [31,32]. The effect of DEX on morphology, chemical structure and mechanical and biological properties of the PHBHV electrospun scaffolds was evaluated. Fibrous PHA meshes with specific characteristics can promote controlled drug release for transdermal/tympanic patches and be produced by electrospinning as a versatile method [33]. Kundrat et al. demonstrated the feasibility of electrospun PHB meshes serving as delivery systems for Levofloxacin, used as a model drug, with PHB concentration and the solvent composition controlling the morphology of the electrospun meshes [34].

Bead formation during the electrospinning process is a common phenomenon that can change fiber properties [35]. When selecting appropriate electrospinning conditions, and most importantly, a suitable solvent system, PHBHV ultrafine fibers with a homogeneous morphology, a relatively uniform diameter and with a few amounts of beads were formed, consistently with previous studies [36]. The presence of DEX (10% w%) slightly increased fiber size (from 0.93 ± 0.23 µm of plain PHBHV to 1.11 ± 0.44 µm of DEX-loaded PHBHV). Moreover, DEX increased the number of beads within the scaffold structure. This might be attributed to a conductivity increase of the polymeric solution due to the addition of DEX. It can be hypothesized that when DEX is mixed with PHBHV, it may aggregate within these beads and they were distributed within fibrous scaffolds [37]. Bead formation in the presence of DEX are considered by some authors to act as drug storage integrated with the electrospun fibers mesh. The phenomena driving drug release are diffusion (i.e., in the first months) followed by the biodegradation of the fibers, which ultimately enables the entrapped drug to be released in a controlled manner from these storages [37]. In a previous study, we investigated the slow degradation of PHBHV fibers loaded with olive oil extract [38]. After 28 days, PHBHV mass loss was ~3.5% in PBS, also in addition of metalloproteinase 9, and the very first signs of surface erosion started to be visible [38]. Therefore, we considered electrospun PHBHV fiber meshes suitable for being in contact with inflamed tissues due to their durability and capability of releasing bioactive agents [36,38]. Electrospinning is a suitable technology to produce ultrafine PHBHV fibers, wherein indeed no significant difference was observed with FTIR in the chemical structure of electrospun fibers in comparison to polymer powder. The main characteristic band of DEX at 1660 cm^−1^ was clearly observed in DEX-loaded PHBHV electrospun fiber. The results are in line with previous studies that reported the entrapment of DEX within electrospun fibers made of other polymers [37].

Indeed, Gaharwaret et al. reported on how to utilize a beaded fibrillar scaffold made of PEOT/PBT, a polyether–ester multiblock copolymer, to facilitate the encapsulation and sustained release of DEX for directing the differentiation of human mesenchymal stem cells. By integrating the amphiphilic beads into the fibrillar scaffolds, they were able to create depots for a sustained drug release. Because of the entrapment of DEX within the beaded structure, the drug was released over a period of 28 days in a sustained manner [37].

PLGA is one of the most studied polymers for drug delivery applications in the form of NPs, due to its biocompatibility and well-studied biodegradation behavior [39,40,41]. Electrospray is a one-step, simple, flexible and versatile method for combining different bioactive agents within polymeric NPs, with a high encapsulation yield, which in other spraying methods, like spray-drying, can reach ≥99%, and has some specific advantages in comparison to the other conventional methods, including scale-up potential, good control of particle size and excellent particulate reproducibility [40]. We used electrospray to produce PLGA NPs as a drug carrier. A half decrease in NP size was observed consequently to RHO addition, from 774 ± 182 nm in plain PLGA to 303 ± 89 nm in RHO-loaded PLGA NPs. During electrospray, a conductive liquid jet breaks up into monodisperse droplets under the influence of an electrical field [40]. In this process, the high evaporation rate of solvent, the high mobility of polymer chains and solution conductivity, as well as decreases solution viscosity, all contribute to a decrease in particle size [20]. In our case, NP size reduction could be attributed to a possible reduction in solution viscosity, or an increase in the conductivity of the polymeric solution due to the presence of RHO [42,43].

PLGA NPs and RHO-loaded PLGA NPs with smooth and roundish surface morphology were successfully electrosprayed on the surface of plain and DEX-loaded PHBHV fiber meshes. A higher population of NPs on the surface of DEX-loaded PHBHV fibers in comparison to the plain ones could be attributed to an increase in the conductivity during electrospray process due to the presence of DEX inside the fibers. We also showed that the electrospray technique is a proper method to produce RHO-loaded NPs since the main characteristic picks of RHO were observed in the spectrum of RHO-loaded PLGA NPs, without significant change in PLGA chemical structure. The effect of DEX on the mechanical properties of electrospun PHBHV fibers was also shown. Sombatmankhong et al. reported the mechanical properties of electrospun fibers based on PHB, PHBV and their blend, and concluded that improved mechanical properties were observed in the blended fiber mats in comparison to those of the plain fiber ones [6]. Our results highlighted that the presence of DEX inside the fibers influenced the mechanical properties of the mesh by reducing the Young’s modulus. Our findings suggested that the DEX acted as a plasticizer inside the fibers without substantially modifying the structure of the PHBHV polymeric matrix [28]. A similar behavior was observed by combining electrospinning with electrospray modes, also in plain PHBHV fiber meshes, which could be attributed to the recently formed fibers interacting with the PLGA NP solvent.

The addition of DEX within the PHBHV electrospun fibers enabled the development of a patch that can facilitate a controllable DEX release. Various factors, such as fibrous mesh structure, polymer composition, hydrophilicity, drug loading capacity, polymer degradation behavior and polymer/drug interactions affect the release of drug or bioactive molecules from the polymeric electrospun meshes [44]. Due to a larger surface area, electrospun patches have a faster, yet still controlled, drug release characteristic than solid slabs [45]. Polymer/water interaction also plays an important role in drug release profiles [37]. Our results showed that a gradual and controlled release of DEX from the electrospun patch occurred over the whole period of the analysis (i.e., 28 days). Moreover, it was observed that the release tended to continue for longer times, since the release curve had not yet reached the equilibrium at 28 days, thus enabling a means for long-term drug delivery, which could be attributed to the hydrophobic nature of PHBHV copolymer, in which drug release is affected by diffusion, followed by hydrolytic biodegradation, which in vitro started around 4 weeks [36]. Many conditions would ideally require a longer yet optimal transdermal therapy than currently available solutions. As an example, idiopathic sudden sensorineural hearing loss is an inner ear pathology that is treated with DEX and can cause permanent deafness if unsuccessfully cured. Intra-tympanic injections offer a promising alternative with patients recurrently treated in the hospital [46]. Having a tympanic patch that can release DEX over a prolonged time after a single application would reduce hospital costs, as well as pain and distress for the patients. Among others, topical DEX is a valid therapy for oral lichen planus [47], which could benefit for a long-lasting patch in the buccal cavity.

It was also displayed that locating PLGA NPs at the surface of PHBHV fibers resulted in a lower percentage of RHO being released in comparison to RHO-loaded PLGA NPs, due to the lower retention of NPs by the fibers for the same electrospray time, which can depend upon an insulating layer generated by the copolymer fibers on the positively charged collector, and would need an optimization of the set-up for the drug chosen in place of RHO. It is evident that the DL capacity of this type of systems is mainly in charge of the electrospun fibers, but the other drug(s) loaded in the NPs could be those necessary at a low dose for an immediate action.

Finally, we tested the cytocompatibility of the produced scaffold with HDFs, in view of possible applications in diseases derived from prolonged and acute inflammatory states, such as those deriving from atopic dermatitis and sudden hearing loss, among others [48,49]. In these systems, the controlled release of DEX by the patch over a long period of time is essential, and optimal tolerance from the surrounding tissues is required. We used HDFs, as they can be a valuable model both for the skin and for the eardrum and cultured them on the patches for 8 days to assess the effect of burst release. The PHBHV scaffold resulted cytocompatible towards HDFs, which adhered on the surface of the mesh, and the presence of DEX had a favorable effect on HDF viability and metabolic activity, as also described by other investigators [50,51]. RHO is reported to reduce collagen synthesis by lip fibroblasts with an unspecific mechanism and cell damage, as no relevant cell death is reported [52]. In our study, RHO was used as a drug model, since its release can be accurately monitored. Metabolic activity and cell viability were comparable in RHO-loaded with plain PLGA NP/PHBHV fiber counterparts, which can be due to the low number of NPs incorporated by the fiber meshes. Other drugs or biomolecules could be used instead of RHO, such as growth factors and antioxidants, to support diverse pathology resolution.

Having an all-in-one method to produce transdermal/tympanic multi-nanodrug delivery patches would advance these TDDSs to enter the market and improve the treatment of skin and ear diseases, not only by a pharmacological action of the drugs, but also by a regenerative action of the fiber scaffolds [36,53].

## 5. Conclusions

Electrospun PHBHV fiber meshes incorporating DEX, combined with electrosprayed PLGA NPs incorporating RHO, the latter as a model drug, were shown as a dual-nanodrug delivery system, potentially useful as skin or tympanic patch, which can represent an all-in-one platform for multi-nanodrug delivery systems. Differently from other applications, in which the NPs are pre-produced with a conventional method and then electrosprayed as a suspension, here, drug-loaded PLGA NPs were produced along with the deposition on the electrospun fibers, which reduced the necessary equipment to fabricate the whole patch to a single equipment, i.e., the electrospinning apparatus. The effect of drug entrapment on morphology, chemical structure and mechanical and biological properties of the fiber meshes was investigated. Thanks to a beaded structure, DEX-loaded fibrous meshes showed a sustained release of DEX over 28 days. RHO-loaded PLGA NPs were successfully produced via electrospray and deposited on the surface of PHBHV fibers, even though in a reduced quantity with respect to the absence of fibers on the collector. HDFs were able to grow and be viable for 8 days on the patches. The produced NP/ultrafine fiber scaffolds showed good potential to act as a carrier for the delivery of multiple bioactive agents in controlled manners in patches for TDDs, including transdermal/tympanic applications.

## Figures and Tables

**Table 1:** Young’s moduli of the different types of PHBHV fiber meshes (*n* = 3) and statistical comparisons; strain ranges used for Young’s moduli calculations due to slipping (related to Figure 5 as a representative example).

#	Sample	Young’s Modulus (MPa)	Strain Range (%)	*p*-Value
1	PHBHV fibers	0.65 ± 0.08	0.05–0.20	<0.05 (#1 vs. #2, #3, #4)
2	DEX-loaded PHBHV fibers	0.18 ± 0.05	0.00–0.20	n.s. (vs. #3, #4)
3	PHBHV fibers + RHO-loaded PLGA NPs	0.14 ± 0.03	0.50–1.00	n.s. (vs. #2, #4)
4	DEX-loaded PHBHV fibers + RHO-loaded PLGA NPs	0.10 ± 0.03	0.45–0.80	n.s. (vs. #2, #3)

n.s. = not statistically significant.

## Data Availability

Data are available upon request to the corresponding authors.

## Supplementary Materials

The following supporting information can be downloaded at: https://www.mdpi.com/article/10.3390/polym15173494/s1, Figure S1: Characterization of human dermal fibroblasts (HDFs); Table S1. Statistical analysis results (*p*-values) for the comparisons between HDFs and the different fiber samples: * *p* < 0.05; ** *p* < 0.001; *** *p* < 0.0001.
