# Supplementary material for: A Straightforward Method to Produce Multi-Nanodrug Delivery Systems for Transdermal/Tympanic Patches Using Electrospinning and Electrospray
Source: Polymers (Basel). 2023 Aug 22;15(17):3494. doi: 10.3390/polym15173494 (PMC10490036; doi:10.3390/polym15173494)
Supplement: Supplementary file 1 [file polymers-15-03494-s001.zip › polymers-2421814-supplementary.pdf]

polymers

MDPI

Supplementary

A Straightforward Method to Produce Multi-Nanodrug Delivery Systems for Transdermal/Tympanic Patches Using Electrospinning and Electrospray

Bahareh Azimi [1,2,*], Claudio Ricci [1,2,3], Teresa Macchi [3], Cemre Günday [4], Sara Munafò [1,2], Homa Maleki [5], Federico Pratesi [3], Veronika Tempesti [2], Caterina Cristallini [2,6], Luca Bruschini [7], Andrea Lazzeri [1,2,6], Serena Danti [1,2,6,*,†] and Nazende Günday-Türeli [4,†]

1 Department of Civil and Industrial Engineering, University of Pisa, Largo L. Lazzarino 2, 56122 Pisa, Italy; claudio.ricci@unipi.it (C.R.); sara.munafo6@gmail.com (S.M.); andrea.lazzeri@unipi.it (A.L.)
2 Consorzio Interuniversitario Nazionale per la Scienza e Tecnologia dei Materiali (INSTM), via G. Giusti 9, 50121 Florence, Italy; v.tempesti@outlook.it (V.T.); caterina.cristallini@cnr.it (C.C.)
3 Department of Translational Researches and New Technologies in Medicine and Surgery, via Savi 10, 56126 Pisa, Italy; teresa.macchi@med.unipi.it (T.M.); federico.pratesi@unipi.it (F.P.)
4 MyBiotech GmbH, Industriestrasse 1B, 66802 Überherrn, Germany; c.guenday@mybiotech.de (C.G.); n.guenday-tuereli@mybiotech.de (N.G.-T.)
5 Department of Carpet, Faculty of Arts, University of Birjand, Birjand 9717434765, Iran; hmaleki@birjand.ac.ir
6 Institute for Chemical and Physical Processes (IPCF), National Council of Researches (CNR), via G. Moruzzi 1, 56124 Pisa, Italy
7 Department of Surgical, Medical, Molecular Pathology and Emergency Medicine, University of Pisa, via Savi 10, 56126 Pisa, Italy; luca.bruschini@unipi.it
* Correspondence: bahareh.azimi@ing.unipi.it (B.A.); serena.danti@unipi.it (S.D.)
† These authors contributed equally to this work.

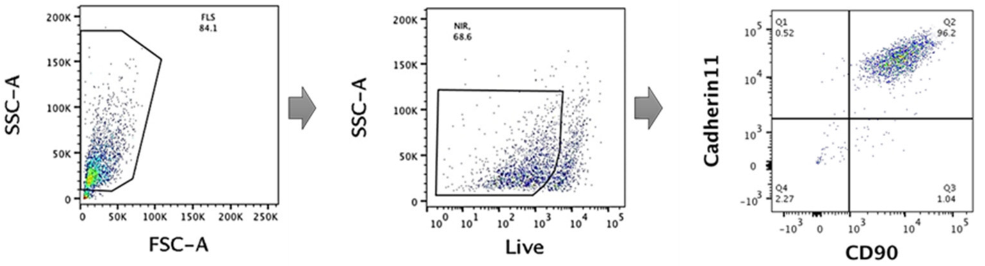

Figure S1. Characterization of human dermal fibroblasts (HDFs). HDFs at passage 3 were stained with Anti-CD90/Thy-1 FITC and anti-Cadherin 11 PE. Cell debris were eliminated in the FSC-SSC gating. Dead cells were excluded after staining with Zombie Aqua fixable dye. As per the expression of CD90 and Cadh-11, HDFs are considered pure at >96%.

Table S1. Statistical analysis results (p-values) for the comparisons between HDFs and the different fiber samples: * $p < 0.05$; ** $p < 0.001$; *** $p < 0.0001$.

**HDFs *versus***	**2 days**	**5 days**	**8 days**
**PHBHV fibers**	***	***	***
**PHBHV fibers + PLGA NPs**	***	***	***
**PHBHV fibers + RHO-loaded PLGA NPs**	***	***	***

**DEX-loaded PHBHV fibers**	*	***	**
**DEX-loaded PHBHV fibers + PLGA NPs**	***	***	***
**DEX-loaded PHBHV fibers + RHO-loaded PLGA NPs**	***	***	***
